# Exploration of errors in variance caused by using the first-order approXimation in Mendelian randomization

**DOI:** 10.5808/gi.21060

**Published:** 2022-03-31

**Authors:** Hakin Kim, Kunhee Kim, Buhm Han

**Affiliations:** 1 Interdisciplinary Program of Bioengineering, Seoul National University College of Engineering, Seoul 08826, Korea; 2 Department of Biomedical Sciences, Seoul National University College of Medicine, Seoul 03080, Korea; 3 Department of Bioinformatics and Computational Biology, The University of Texas MD Anderson Cancer Center, Houston, TX 77030, USA; 4 UTHealth Graduate School of Biomedical Sciences, The University of Texas MD Anderson Cancer Center, Houston, TX 77030, USA

**Keywords:** computer simulation, delta method, Mendelian randomization analysis

## Abstract

Mendelian randomization (MR) uses genetic variation as a natural experiment to investigate the causal effects of modifiable risk factors (exposures) on outcomes. Two-sample Mendelian randomization (2SMR) is widely used to measure causal effects between exposures and outcomes via genome-wide association studies. 2SMR can increase statistical power by utilizing summary statistics from large consortia such as the UK Biobank. However, the first-order term approXimation of standard error is commonly used when applying 2SMR. This approXimation can underestimate the variance of causal effects in MR, which can lead to an increased false-positive rate. An alternative is to use the second-order approXimation of the standard error, which can considerably correct for the deviation of the first-order approXimation. In this study, we simulated MR to show the degree to which the first-order approXimation underestimates the variance. We show that depending on the specific situation, the first-order approXimation can underestimate the variance almost by half when compared to the true variance, whereas the second-order approXimation is robust and accurate.

## Introduction

It is important to understand the causality between two phenotypes to uncover the pathogenesis of diseases. Some strategies eXist for assessing causality in epidemiological studies. Mendelian randomization (MR) is a technique that uses genetic variants as instrumental variables (IVs) to estimate the causal effect of an exposure on an outcome [1]. In accordance with Mendel’s laws of inheritance, alleles are randomly inherited from parents. Therefore, the genotypes of offspring can be considered independent of confounding factors. Furthermore, the fact that genotypes are fixed and are not affected by phenotypes obviates the reverse causation problem. For these reasons, genetic variants naturally meet many of the basic assumptions of IVs.

Summary statistics released from large genome-wide association studies recently began to facilitate MR by providing exposure effect sizes for multiple genetic variants [2]. The type of MR analysis using an external dataset for quantifying exposure effect is called a two-sample MR design (2SMR). An advantage of 2SMR is that the statistical power can be increased by merging summary statistics from various sources including large consortia such as the UK Biobank [3]. The causal effect between an exposure and an outcome is estimated by the ratio between the reported genetic effect to the exposure in an external dataset and the observed genetic effect to the outcome in the target dataset. Since there are multiple variants, the ratio estimates over multiple variants are usually combined into a single estimate via the inverse-variance weighted method.

In 2SMR, the standard error of the estimated ratio is conventionally approXimated by the first-order term from the delta method. As stated by Thomas et al. [4], however, this approXimation can lead to an underestimation of the variance. This underestimation can lead to both increased power and an increased false-positive rate (FPR). An alternative is to use the second-order approXimation of the standard error, which can considerably correct for the deviation of the first-order approXimation.

In this study, we extensively simulate MR to show the impact of this first-order approXimation on the FPR and power of MR. We simulate several different situations to evaluate which study design parameters affect the errors of the first-order approXimation, and also compare the errors of the first-order approXimation to those of the second-order approXimation.

## Methods

### Genetic variants as instrumental variables

**Fig. 1.**
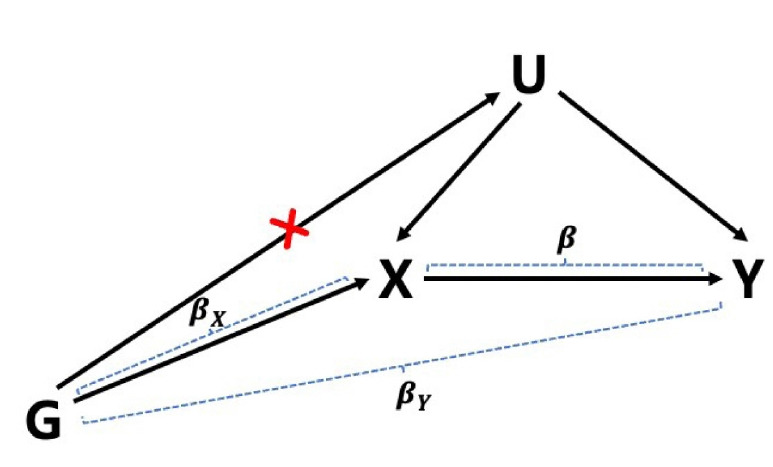
Diagram depicting the relationships of instrumental variable (IV), exposure, outcome and confounder. Under the assumption of Mendelian randomization, IV should not affect the confounder (red cross). G denotes an IV which is SNP in our case, X denotes an exposure, Y denotes an outcome and U denotes the confounder such as smoking.

Genetic variants such as single-nucleotide polymorphisms (SNPs) have several properties that make them appropriate as an instrument of exposure. The random inheritance of the alleles makes the genotype distribution independent of socio-economic factors and lifestyle factors such as income [5]. Inherited alleles are not changed from birth by diseases or conditions, except in rare cases of somatic mutations. However, some assumptions still need to be satisfied to ensure the validity of a genetic variant as an IV (Fig. 1). Three basic assumptions must hold for a genetic variant to be used as an IV for MR [6].

IV1. The genetic variant is associated with the exposure.

IV2. The genetic variant influences the outcome only through the exposure.

IV3. The genetic variant is independent of confounding factors affecting the exposure-outcome relationship.

Whether these assumptions are satisfied in various conditions has been discussed elsewhere [7]. Herein, we simply accept these assumptions and proceed to the description of MR.

### Basic model of MR and the first-order approXimation of variance

In this section, we describe the basic model of MR along with the commonly used first-order variance approXimation (Fig. 1). Let G be an IV (e.g., a SNP), X be an exposure such as body mass index, and Y be an outcome, such as disease. We can set the relationships between variables (G, X, and Y) via a linear regression model.


$$X|G_{X}\;=\;\beta_{X0}\;+\;\beta_{X}G_{X}\;+\;\varepsilon_{X}$$

$$Y|G_{Y}\;=\;\beta_{Y0}\;+\;\beta_{Y}G_{Y}\;+\;\varepsilon_{Y}$$

If we assume that all IV assumptions are satisfied, then *β*_X_≠0 because of IV1 and *β*_Y_ = *β*_X_×*β* because of IV2 and IV3. That is, G (Fig. 1) affects Y (outcome) only through X (exposure). It is assumed that the error terms ε_X_ and ε_Y_ follow normal distributions and are independent in the case of 2SMR of two disjoint samples. Even in the case of two non-overlapping samples, a report has stated the sample correlation between $\hat{\beta_{X}}$ and $\hat{\beta_{Y}}$ can be ignored [8]. The ratio estimate $\hat{\beta }={\hat{\beta }}_{X}/{\hat{\beta }}_{Y}$ reflects the causal effect between exposure and outcome, and is consistent asymptotically.

To test whether *β*≠0, it is essential to obtain the variance estimate of $\hat{\beta }$. The commonly used first-order approXimation is $\mathrm{Var}(\hat{\beta })\approx \frac{\mathrm{Var}(\hat{\beta_{Y}})}{{\hat{\beta_{X}}}^{2}}$. The first-order approXimation method involves treating the denominator ${\hat{\beta }}_{X}$ as a constant. However, because of the innate uncertainty in ${\hat{\beta }}_{X}$, we can expect that $\frac{\mathrm{Var}(\hat{\beta_{Y}})}{{\hat{\beta_{X}}}^{2}}$ tends to underestimate the true variance of $\hat{\beta }$.

### The second-order approXimation method of variance of estimated causal effects

Thomas et al. [4] suggested a second-order approXimation of the variance of $\hat{\beta }$. With the delta method, one can approXimate the variance of causality $\hat{\beta }$ as follows.


$$\mathrm{Var}(\hat{\beta })\;\approx \;\frac{\mathrm{Var}(\hat{\beta_{Y}})}{{\hat{\beta_{X}}}^{2}}-2\frac{\hat{\beta_{Y}}}{{\hat{\beta_{X}}}^{3}}\mathrm{Cov}(\hat{\beta_{X}},\;\hat{\beta_{Y}})+\;\frac{{\hat{\beta_{Y}}}^{2}}{{\hat{\beta_{X}}}^{4}}\mathrm{Var}(\hat{\beta_{X}})$$

Since we use different samples (2SMR), we can set $\mathrm{Cov}(\hat{\beta_{X}},\;{\hat{\beta }}_{Y})=0$, as X and Y are from non-overlapping samples. Therefore, we obtain the following approXimation.


$$\mathrm{Var}(\hat{\beta })\;\approx \;\frac{\mathrm{Var}(\hat{\beta_{Y}})}{{\hat{\beta_{X}}}^{2}}+\frac{{\hat{\beta_{Y}}}^{2}}{{\hat{\beta_{X}}}^{4}}\mathrm{Var}(\hat{\beta_{X}})$$

The second term is always positive. Therefore, if researchers use only the first term from this approXimation for the variance, this can lead to an underestimation of the standard error.

### Simulation design

We designed simulations to evaluate the magnitude of error in the first-order approXimation method. We assumed specific true values for *β* and *β*_X_, which also gave us the true value of *β*_Y_=*β*×*β*_X_. We assumed the intercepts *β*_X0_=0.03 and *β*_Y0_=0.03, and the errors $\sqrt{\mathrm{Var}(\varepsilon_{X})}=\mathrm{sd}(\varepsilon_{X})=0.3$ and $\sqrt{\mathrm{Var}(\varepsilon_{Y})}=\mathrm{sd}(\varepsilon_{Y})=0.3$ . We independently generated genotypes (SNP alleles) G_X_ and G_Y_, which are composed of 0, 1, and 2 from the distribution Binomial(2, MAF), where MAF denotes the minor allele frequency. We generated (X|G_X_, Y|G_Y_) by adding noise with mean 0 and variance (Var(ε_X_), Var(ε_Y_)) to (*β*_X0_+*β*_X_ G_X_, *β*_Y0_+*β*_Y_ G_Y_). Then we obtained $\hat{\beta_{X}}$ and $\hat{\beta_{Y}}$ via simple linear regression. We can expect $\hat{\beta_{X}}$ and $\hat{\beta_{Y}}$ to be randomly distributed by


$$\hat{\beta_{X}}\sim N(\beta_{X},\;\frac{\mathrm{Var}(\varepsilon_{X})}{{\mathrm{SS}}_{{\mathrm{GG}}_{X}}})\;\hat{\beta_{Y}}\sim N\left(\beta_{Y},\;\frac{\mathrm{Var}\left(\varepsilon_{Y}\right)}{{\mathrm{SS}}_{{\mathrm{GG}}_{Y}}}\right)$$

${\mathrm{SS}}_{{\mathrm{GG}}_{X}}=\Sigma G_{\mathrm{Xi}}^{2}-\frac{(\Sigma G_{\mathrm{Xi}}{)}^{2}}{N_{x}}$ and ${\mathrm{SS}}_{{\mathrm{GG}}_{Y}}=\Sigma G_{\mathrm{Yi}}^{2}-\frac{(\Sigma G_{\mathrm{Yi}}{)}^{2}}{N_{y}}$. where N_x_ is the size of the reference dataset used in 2SMR and N_y_ is the size of the target sample.

To approXimate Var( $\hat{\beta }$ ), we can use either the first-order or the second-order approximation:

First-order: $\mathrm{Var}(\hat{\beta })\approx \frac{\mathrm{Var}(\hat{\beta_{Y}})}{{\hat{\beta_{X}}}^{2}}$

Second-order: $\mathrm{Var}(\hat{\beta })\approx \frac{\mathrm{Var}(\hat{\beta_{Y}})}{{\hat{\beta_{X}}}^{2}}+\frac{{\hat{\beta_{Y}}}^{2}}{{\hat{\beta_{X}}}^{4}}\mathrm{Var}(\hat{\beta_{X}})$

Our simulation allowed us to empirically obtain a very accurate estimate of Var( $\hat{\beta }$ ) by repeating the simulation many times (we set the number of simulations as 100,000 in our study) with the same assumptions and calculating the observed variance of $\hat{\beta }$ . This allowed us to compare the first and second-order approXimations to the empirically obtained values.

We provide the R script code to run the entire simulation pipeline as Supplementary Data.

## Results

We performed empirical simulations to compare the two types of analytical approXimations: the classical way, in which only the first-order term is used, and the recently suggested way [4], which includes up to the second-order term. We also obtained an accurate estimate of the variance by empirically repeating simulations 100,000 times. Assuming that the empirically obtained variance is the gold standard, we calculated the ratio of the estimated variance to the gold standard.

**Fig. 2.**
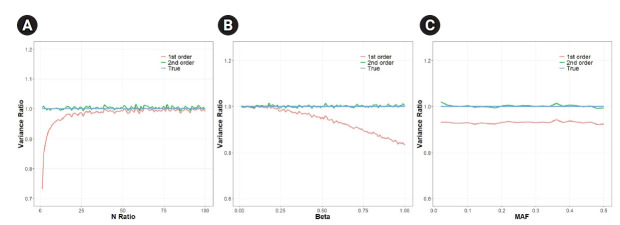
The ratio of the approXimation of the variance of causal effect estimate to the true value. (A) We varied the N-ratio (N_x_/N_y_) value from 1 to 100 assuming N_x_ = 200,000, *β*_X_ = 0.02, *β* = 0.6, and minor allele frequency (MAF) = 0.2. (B) We varied the value *β*, i.e. the ratio of *β*_X_ and *β*_Y_ from 0.01 to 1 assuming *β*_X_ = 0.02, N_x_ = 200,000, N_y_ = 10,000 and MAF = 0.2. (C) We varied the MAF from 0.02 to 0.5 assuming *β* = 1, *β*_X_ = 0.02, N_x_ = 200,000 and N_y_ = 10,000. The true value was estimated by empirical simulations (Nsim = 100,000).

In our simulations, we varied multiple parameters. We varied the N-ratio (N_x_/N_y_), we also varied *β* (the magnitude of causal effect) and MAF. Fig. 2 shows that the analytical approXimation that contained variance up to the second-order term was almost as accurate as the empirical estimate, whereas the first-order approXimation method was often largely inaccurate depending on the situation.

Fig. 2A shows that the error due to the first-order approXimation decreased as the number of individuals (N_y_) decreased from 200,000 to 2,000 (as the N-ratio increased from 1 to 100). The ratio was 0.84 when N_y_ was 100,000, which is equal to N_x_/2 (N-ratio=2). The ratio rose to 0.99 when N_y_ was 2,000 (N-ratio = 100). The mean of the ratios was 0.98, which translates to a reduced SE($\hat{\beta }$) by $\sqrt{0.98}=0.999$ times in the first-order approXimation. Fig. 2B shows that the errors increased when the actual causal effect (*β*) between the exposure and outcome increased from 0.01 to 1. Therefore, if there is not a strong causal effect between the exposure and outcome in MR, the error from the first-order approXimation would be small. The mean of the ratios of the first-order approXimation was 0.93. Fig. 2C shows that, interestingly, the ratio appeared to be independent of the MAF of the variant. The mean of the ratios in this simulation was 0.93 in the first-order case.

**Fig. 3.**
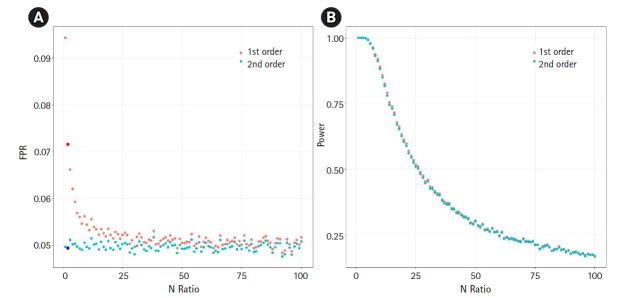
The scatter plots of false-positive rate (FPR) and statistical power. (A) The scatter plot of relationship between the N-ratio in the simulation of Fig. 2A and FPR. If N-ratio=2 (N_y_ = 100,000 and N_x_ = 200,000), the FPRs were 0.071 (the dark red colored large dot) for the first-order approXimation and 0.049 (the dark blue colored large dot) for the second-order approXimation. To calculate FPR values, we generated 100,000 samples from the null hypothesis of no causal effect. (B) The scatter plot of relationship between the N-ratio the power. We generated 100,000 samples with *β* = 0.6 which is the causal effect of the exposure on the outcome.

We then analyzed the impact of the underestimated variance. If the variance is underestimated, the FPR can increase. We assumed the null hypothesis of no causal effect and generated 100,000 samples under an environment equivalent to that of Fig. 2A. We calculated the FPR based on the significance threshold of α=0.05. Fig. 3A shows the relationship between the N-ratio and the FPR. Notably, when the variance was underestimated by a factor of 0.84, as shown in Fig. 2A (for the case of an N-ratio = 2—that is, N_y_ = 100,000 and N_x_ = 200,000), the FPR of the first-order approXimation method increased to 0.071 (the dark red colored large dot in Fig. 3A), while the FPR of the second-order approXimation method was 0.049 (the dark blue colored large dot in Fig. 3A), corresponding to approXimately 0.7 times that of the first-order case. The average FPR in the second-order approXimation method was 0.049, whereas the average FPR in the first-order approXimation was 0.052. These findings indicate that the second-order approXimation can be a good choice to prevent inflation of the FPR.

We also analyzed the statistical power (Fig. 3B). Since the variance of $\hat{\beta }$ is underestimated, the first-order approXimation method may also tend to increase the power (or underestimate the false-negative rate). To compare the powers of the first and the second-order approXimation methods, we generated 100,000 samples under an environment equivalent to that of Fig. 2A, with *β* = 0.6, which denotes the causal effect of the exposure on the outcome. Under this setting, the power of the first-order approXimation was similar to that of the second-order approXimation (on average 1.01 times greater).

## Discussion

In this study, we performed simulations to evaluate the errors in the variance estimate of causal effects in 2SMR. We simulated a range of study parameters and showed that the commonly used first-order approXimation can be inaccurate depending on the situation, while the second-order approXimation is consistently accurate. We then showed that the underestimated variance can lead to a significant increase in the FPR.

In our simulations, the variance errors due to the first-order approXimation were dependent on parameters such as the N-ratio and the *β*-ratio. When the number of samples in the target study increased while the number of samples in the external dataset for exposure association was fixed, the errors became larger. This suggested that in future studies, a larger study size may correspond to increased error from the first-order approXimation method. Furthermore, as the true causal effect increased, so did errors. Interestingly, the errors appeared to be independent of the MAF.

In this study, we simply assumed the use of a single SNP as an IV in 2SMR. The causal effect between an exposure and an outcome is usually obtained by merging the ratio per variant (*β*) via the inverse-variance weighted method over a large number of variants. In this extended multi-variant model, we expect that the variance of the final estimate will also be affected by the errors induced by the first-order approXimation, because the ratio for all variants is affected regardless of MAF. Then, the standard error of the causal effect, $\hat{\beta }$, would be dependent on the same parameters (N-ratio and the magnitude of beta) as in the extended model. Some other issues, such as linkage disequilibrium and pleiotropy, should also be addressed in the extended multivariate model.

Overall, our study suggests that the use of the second-order approXimation is always preferable, since it provides an accurate estimate of the variance regardless of the situation. However, when the IV-exposure association is much greater than the IV-outcome association (i.e., *β* is very small), we observed no significant difference between the first- and second-order approXimations. Therefore, we expect that whether one must apply the second-order approXimation to avoid an increased FPR will depend on many factors, including the actual range of *β*.

## Supplementary Materials

Supplementary Data. Our simulation was run by R. For visualization, 'ggplot2' package is required.
