# Supplementary material for: Exploration of errors in variance caused by using the first-order approXimation in Mendelian randomization
Source: Genomics Inform. 2022 Mar 31;20(1):e9. doi: 10.5808/gi.21060 (PMC9002003; doi:10.5808/gi.21060)
Supplement: Supplementary Data. — Our simulation was run by R. For visualization, 'ggplot2' package is required. [file gi-21060suppl1.pdf]

Supplementary Fig. 1. Our simulation was run by R. For visualization, 'ggplot2' package is required.

```
library(ggplot2)

## Warning: 패키지 'ggplot2'는 R 버전 4.1.2 에서 작성되었습니다

set.seed(1)

b <- 0.6
nx <- 200000
max <- 100
maf <- 0.2
bx0 <- 0.03
bx1 <- 0.02
sx <- 0.3
nsim <- 100000

by0 <- 0.03
by1 <- b*bx1
sy <- 0.3


rort <- c()
rnrt <- c()
rtrt <- c()

fpr1 <- c()
fpr2 <- c()
fnr1 <- c()
fnr2 <- c()

#1 : second-order
#2 : first-order
for(k in 1:max){
  x_x <- rbinom(nx, 2, maf)
  y_x <- bx0 + bx1*x_x + rnorm(nx, 0, sx)
  sd_b1x_hat <- sx/sqrt(sum(x_x^2)-((sum(x_x))^2)/length(x_x))

  nratio <- k
  ny <- as.integer(nx/nratio)

  x_y <- rbinom(ny, 2, maf)
  y_y <- by0 + by1*x_y + rnorm(ny, 0, sy)
  sd_b1y_hat <- sy/sqrt(sum(x_y^2)-((sum(x_y))^2)/length(x_y))

  bxhat <- rnorm(nsim, bx1, sd_b1x_hat)
  byhat <- rnorm(nsim, by1, sd_b1y_hat)
  bhat <- byhat/bxhat

  varbhat1 <- mean((sd_b1y_hat^2)/(bxhat^2)+((sd_b1x_hat^2)*(byhat^2))/(bxhat
```

```
^4))
  varbhat2 <- mean((sd_b1y_hat^2)/(bxhat^2))

  rort[k] <- varbhat2/var(bhat)
  rnrt[k] <- varbhat1/var(bhat)
  rtrt[k] <- 1

  betas <- rnorm(100000,0, sqrt(var(bhat)))
  fpr2[k] <- mean((pnorm(abs(betas), sd=sqrt(varbhat2),lower.tail = F)*2)<=0.
05)
  fpr1[k] <- mean((pnorm(abs(betas), sd=sqrt(varbhat1),lower.tail = F)*2)<=0.
05)

  betas_causal <- rnorm(100000,b, sqrt(var(bhat)))
  fnr2[k] <- mean((pnorm(abs(betas_causal), sd=sqrt(varbhat2),lower.tail = F)
*2)>0.05)
  fnr1[k] <- mean((pnorm(abs(betas_causal), sd=sqrt(varbhat1),lower.tail = F)
*2)>0.05)

}

mydat <- data.frame(Proportion = c(rnrt, rort, rep(1,max)), Class=c(rep('2nd
order',max), rep('1st order',max),rep('True',max)), N_Ratio=c(1:max,1:max,1:m
ax))




#Figure 2a
ggplot(mydat, aes(x=N_Ratio)) +
  geom_line(aes(y=Proportion, col=Class), size=1.3) +
  theme_bw()+
  theme(axis.text.x = element_text(angle = 0, vjust=0.5, size = 15),
        axis.text.y = element_text(angle = 0, vjust=0.5, size = 15),
        panel.grid.minor = element_blank()) +
  coord_cartesian(xlim = c(1,max), ylim = c(0.7,1.2)) +
  ggtitle("(a)\n") +
  labs(x='N Ratio', y='Variance Ratio') +
  theme(axis.title=element_text(size=20,face="bold"),
        plot.background = element_rect(),
        legend.position=c(.8, .88),
        legend.text=element_text(size=18),
        legend.title=element_text(size=20),
        plot.title=element_text(size=25))

mydat1 <- data.frame(c(fpr2,fpr1),c(1:max,1:max),rep(c('1st order','2nd order
'),each=length(rort)))
colnames(mydat1) <- c('FPR','N_ratio','Class')
mydat2 <- data.frame(c(fnr2,fnr1),c(1:max,1:max),rep(c('1st order','2nd order
```

```
'),each=length(rort)))
colnames(mydat2) <- c('FNR','N_ratio','Class')
mydat3 <- data.frame(1-c(fnr2,fnr1),c(1:max,1:max),rep(c('1st order','2nd ord
er'),each=length(rort)))
colnames(mydat3) <- c('Power','N_ratio','Class')



#Figure 3
ggplot(mydat1, aes(x=N_ratio)) +
  geom_point(aes(y=FPR,col=Class), size=2) +
  labs(x='N Ratio', y='FPR') +
  theme_bw()+
  ggtitle("(a)\n") +
  theme(axis.text.x = element_text(angle = 0, vjust=0.5, size = 15),
        axis.text.y = element_text(angle = 0, vjust=0.5, size = 15),
        panel.grid.minor = element_blank())+
  theme(axis.title=element_text(size=20,face="bold"),
        plot.background = element_rect(),
        legend.position=c(.8, .88),
        legend.text=element_text(size=18),
        legend.title=element_text(size=20),
        plot.title=element_text(size=25))+
  geom_point(mapping =aes(x = N_ratio[2], y = FPR[2]), cex=3,col='red')+
  geom_point(mapping =aes(x = N_ratio[102], y = FPR[102]), cex=3,col='blue')

ggplot(mydat3, aes(x=N_ratio)) +
  geom_point(aes(y=Power,col=Class), size=2) +
  labs(x='N Ratio', y='Power') +
  theme_bw()+
  ggtitle("(b)\n") +
  theme(axis.text.x = element_text(angle = 0, vjust=0.5, size = 15),
        axis.text.y = element_text(angle = 0, vjust=0.5, size = 15),
        panel.grid.minor = element_blank())+
  theme(axis.title=element_text(size=20,face="bold"),
        plot.background = element_rect(),
        legend.position=c(.8, .88),
        legend.text=element_text(size=18),
        legend.title=element_text(size=20),
        plot.title=element_text(size=25))+
  geom_point(mapping =aes(x = N_ratio[2], y = Power[2]), cex=3,col='red')+
  geom_point(mapping =aes(x = N_ratio[102], y = Power[102]), cex=3,col='blue')
```

```r
#Figure 2b

set.seed(1)

nx <- 200000
max <- 100
maf <- 0.2
bx0 <- 0.03
bx1 <- 0.02
sx <- 0.2

by0 <- 0.03
by1 <- b*bx1
sy <- 0.1



rort <- c()
rnrt <- c()
rtrt <- c()
b_set <- c()
nsim <- 100000


nratio <- 20
ny <- as.integer(nx/nratio)

for(k in 1:max){
  b <- 1/100 * k
  by1 <- b*bx1

  x_x <- rbinom(nx, 2, maf)
  y_x <- bx0 + bx1*x_x + rnorm(nx, 0, sx)
  sd_b1x_hat <- sx/sqrt(sum(x_x^2)-((sum(x_x))^2)/length(x_x))

  x_y <- rbinom(ny, 2, maf)
  y_y <- by0 + by1*x_y + rnorm(ny, 0, sy)
  sd_b1y_hat <- sy/sqrt(sum(x_y^2)-((sum(x_y))^2)/length(x_y))

  bxhat <- rnorm(nsim, bx1, sd_b1x_hat)
  byhat <- rnorm(nsim, by1, sd_b1y_hat)
  bhat <- byhat/bxhat

  varbhat1 <- mean(sd_b1y_hat^2/(bxhat^2)+((sd_b1x_hat^2)*(byhat^2))/(bxhat^
4))
  varbhat2 <- mean(sd_b1y_hat^2/(bxhat^2))

  rort[k] <- varbhat2/var(bhat)
```

```
  rnrt[k] <- varbhat1/var(bhat)
  rtrt[k] <- 1
  b_set[k] <- b
}

mydat <- data.frame(Proportion = c(rnrt, rort, rep(1,max)), Class=c(rep('2nd
order',max), rep('1st order',max),rep('True',max)), b_set=c(b_set,b_set,b_se
t))
ggplot(mydat, aes(x=b_set)) +
  geom_line(aes(y=Proportion, col=Class), size=1.3) +
  theme_bw()+
  theme(axis.text.x = element_text(angle = 0, vjust=0.5, size = 15),
        axis.text.y = element_text(angle = 0, vjust=0.5, size = 15),
        panel.grid.minor = element_blank()) +
  coord_cartesian( ylim = c(0.6,1.25)) +
  ggtitle("(b)\n") +
  labs(x='Beta', y='Variance Ratio') +
  theme(axis.title=element_text(size=20,face="bold"),
        plot.background = element_rect(),
        legend.position=c(.8, .88),
        legend.text=element_text(size=18),
        legend.title=element_text(size=20),
        plot.title=element_text(size=25))






#Figure 2c

set.seed(1)

b <- 0.6
nx <- 200000
max <- 25

bx0 <- 0.03
bx1 <- 0.02
sx <- 0.2

by0 <- 0.03
by1 <- b*bx1
sy <- 0.1

rort <- c()
rnrt <- c()
rtrt <- c()
```

```
nsim <- 100000
nratio <- 20

ny <- as.integer(nx/nratio)



for(k in 1:max){
  maf <- 0.02 * k
  x_x <- rbinom(nx, 2, maf)
  y_x <- bx0 + bx1*x_x + rnorm(nx, 0, sx)
  sd_b1x_hat <- sx/sqrt(sum(x_x^2)-((sum(x_x))^2)/length(x_x))


  x_y <- rbinom(ny, 2, maf)
  y_y <- by0 + by1*x_y + rnorm(ny, 0, sy)
  sd_b1y_hat <- sy/sqrt(sum(x_y^2)-((sum(x_y))^2)/length(x_y))

  bxhat <- rnorm(nsim, bx1, sd_b1x_hat)
  byhat <- rnorm(nsim, by1, sd_b1y_hat)
  bhat <- byhat/bxhat

  varbhat1 <- mean((sd_b1y_hat^2)/(bxhat^2)+((sd_b1x_hat^2)*(byhat^2))/(bxhat
^4))
  varbhat2 <- mean((sd_b1y_hat^2)/(bxhat^2))

  rort[k] <- varbhat2/var(bhat)
  rnrt[k] <- varbhat1/var(bhat)
  rtrt[k] <- 1

}

mydat <- data.frame(Proportion = c(rnrt, rort, rep(1,max)), Class=c(rep('2nd
order',max), rep('1st order',max),rep('True',max)), N_Ratio=c((1:max)/(2*max),
(1:max)/(2*max),(1:max)/(2*max)))
ggplot(mydat, aes(x=N_Ratio)) +
  geom_line(aes(y=Proportion, col=Class), size=1.3) +
  theme_bw()+
  theme(axis.text.x = element_text(angle = 0, vjust=0.5, size = 15),
        axis.text.y = element_text(angle = 0, vjust=0.5, size = 15),
        panel.grid.minor = element_blank()) +
  coord_cartesian(xlim = c(1/(2*max),1/2), ylim = c(0.6,1.25)) +
  ggtitle("(c)\n") +
  labs(x='MAF', y='Variance Ratio') +
  theme(axis.title=element_text(size=20,face="bold"),
        plot.background = element_rect(),
        legend.position=c(.8, .88),
```

```
         legend.text=element_text(size=18),
         legend.title=element_text(size=20),
         plot.title=element_text(size=25))
```
